# Efficient and accurate greedy search methods for mining functional modules in protein interaction networks

**DOI:** 10.1186/1471-2105-13-S10-S19

**Published:** 2012-06-25

**Authors:** Jieyue He, Chaojun Li, Baoliu Ye, Wei Zhong

**Affiliations:** 1 School of Computer Science and Engineering, Key Lab of Computer Network & Information Integration, MOE, Southeast University, Nanjing, 210018, China; 2 National Key Laboratory for Novel Software Technology, Nanjing University, Nanjing, 210093, China; 3 Division of Mathematics and Computer Science, University of South Carolina Upstate 800 University Way, Spartanburg, SC 29303, USA

## Abstract

**Background:**

Most computational algorithms mainly focus on detecting highly connected subgraphs in PPI networks as protein complexes but ignore their inherent organization. Furthermore, many of these algorithms are computationally expensive. However, recent analysis indicates that experimentally detected protein complexes generally contain Core/attachment structures.

**Methods:**

In this paper, a Greedy Search Method based on Core-Attachment structure (GSM-CA) is proposed. The GSM-CA method detects densely connected regions in large protein-protein interaction networks based on the edge weight and two criteria for determining core nodes and attachment nodes. The GSM-CA method improves the prediction accuracy compared to other similar module detection approaches, however it is computationally expensive. Many module detection approaches are based on the traditional hierarchical methods, which is also computationally inefficient because the hierarchical tree structure produced by these approaches cannot provide adequate information to identify whether a network belongs to a module structure or not. In order to speed up the computational process, the Greedy Search Method based on Fast Clustering (GSM-FC) is proposed in this work. The edge weight based GSM-FC method uses a greedy procedure to traverse all edges just once to separate the network into the suitable set of modules.

**Results:**

The proposed methods are applied to the protein interaction network of S. cerevisiae. Experimental results indicate that many significant functional modules are detected, most of which match the known complexes. Results also demonstrate that the GSM-FC algorithm is faster and more accurate as compared to other competing algorithms.

**Conclusions:**

Based on the new edge weight definition, the proposed algorithm takes advantages of the greedy search procedure to separate the network into the suitable set of modules. Experimental analysis shows that the identified modules are statistically significant. The algorithm can reduce the computational time significantly while keeping high prediction accuracy.

## Background

With the rapid development of technologies to predict protein interactions, huge data sets portrayed as networks have been available. Most real networks typically contain parts in which the nodes are more highly connected to each other than to the rest of the network. The sets of such nodes are usually called clusters, communities, or modules [1-4]. The presence of biologically relevant functional modules in Protein-Protein Interaction (PPI) graphs has been confirmed by many researchers [4,5]. Identification of functional modules is crucial to the understanding of the structural and functional properties of networks [6,7]. There is a major distinction between two biological concepts, namely, protein complexes and functional modules [7]. A protein complex is a physical aggregation of several proteins (and possibly other molecules) via molecular interaction (binding) with each other at the same location and time. A functional module also consists of a number of proteins (and other molecules) that interact with each other to control or perform a particular cellular function. Unlike protein complexes, proteins in a functional module do not necessarily interact at the same time and location. In this paper, we do not distinguish protein complexes from functional modules because the protein interaction data used for detecting protein complex in this work do not provide temporal and spatial information.

Recently, many research works have been conducted to solve the problem of clustering protein interaction networks [8-10]. Some of them are using the graph-based clustering methods for mining functional modules [11,17-20]. These studies are mainly based on the observation that densely connected regions in the PPI networks often correspond to actual protein functional modules. In short, methods proposed in these studies are used to detect densely connected regions of a graph that are separated by sparse regions. Some graph clustering approaches using PPI networks for mining functional modules are introduced in the following. Bader and Hogue [17] proposed the Molecular COmplex Detection (MCODE) algorithm that utilizes connectivity values in protein interaction graphs to mine for protein complexes. The algorithm first computes the vertex weight value from its neighbour density and then traverses outward from a seed protein with a high weighting value to recursively include neighbouring vertices whose weights are above a given threshold. However, since the highly weighted vertices may not be highly connected to each other, the algorithm does not guarantee that the discovered regions are dense. A simultaneous protein interaction network (SPIN) introduced by Jung et al [12] specifies mutually exclusive interactions (MEIs). Taking advantages of the SPINs, SPIN_MCODE has outperformed the plain MCODE method.

Amin et al. [18] proposed a cluster periphery tracking algorithm (DPClus) to detect protein complexes by keeping track of the periphery of a detected cluster. DPClus first weighs each edge based on the common neighbours between two proteins and further weighs nodes by their weighted degree. To form a protein complex, DPClus first selects the seed node having the highest weight as the initial cluster and then iteratively augments this cluster by including vertices one by one, which are out of but closely related with the current cluster. Li et al. [13] modified the DPClus algorithm for identifying protein complexes that have a small diameter (or a small average vertex distance) and satisfy a different cluster connectivity-density property. The performance of such algorithms depends heavily on the quality of the seeds and the criterion of extending clusters.

Adamcsek et al. [19] provided a software called CFinder to find functional modules in PPI networks. CFinder detects the k-clique percolation clusters as functional modules using a Clique Percolation Method (CPM)[20]. In particular, a k-clique is a clique with k nodes and two k-cliques are adjacent if they share (k - 1) common nodes. A k-clique percolation cluster is then constructed by linking all the adjacent k-cliques as a bigger subgraph. Li et al. [14] proposed a new clustering algorithm called IPC-MCE to identify protein complexes based on maximal clique, and then extend all the maximal cliques by adding their neighbourhoods iteratively. Liu et al [15] developed an algorithm called Clustering based on Maximal Cliques (CMC) to discover complexes from the weighted PPI network. CMC first finds maximal cliques from PPI networks, and then removes or merges highly overlapped maximal cliques based on their inter-connectivity. However, CMC generates less number of significant functional modules having P-value less than 1E-5 than the DPClus algorithm in the unweighted PPI network [11]. Wang et al. [16] also developed an algorithm called CP-DR based on the new topological model for identifying protein complexes. Wang's algorithm extended the definition of k-clique community of the CPM algorithm and introduced distance restriction.

Above computational studies mainly focus on detecting highly connected subgraphs in PPI networks as protein complexes but ignore their inherent organization. However, recent analysis indicates that experimentally detected protein complexes generally contain Core/attachment structures. Protein complexes often include cores in which proteins are highly co-expressed and share high functional similarity. And core proteins are usually more highly connected to each other and may have higher essential characteristics and lower evolutionary rates than those of peripheral proteins [26]. A protein complex core is often surrounded by some attachments, which assist the core to perform subordinate functions. Gavin et al.'s work [28] also demonstrates the similar architecture and modularity for protein complexes. Therefore, protein complexes have their inherent organization [26,27,29] of core-attachment. To provide insights into the inherent organization of protein complexes, some methods [21,26,29] are proposed to detect protein complexes in two stages. In the first stage, protein complex cores, as the heart of the protein complexes, are first detected. In the second stage, protein complexes are expanded by incorporating attachments into the protein complex cores. Wu et al. [21] presented a COre-AttaCHment based method (COACH) and Leung et al. also developed an approach called CoreMethod. These approaches are used to detect protein complexes in PPI networks by identifying their cores and attachments separately [29]. To detect cores, COACH performs local search within vertex's neighbourhood graphs while the CoreMethod [29] computes the p-values between all the proteins in the whole PPI networks.

In this paper, a Greedy Search Method based on Core-Attachment structure called GSM-CA is introduced. Comparing with the other methods of core-attachment, the new edge weight calculation method and evaluation criterion for judging a node as a core node or an attachment node are proposed in our GSM-CA method. The GSM-CA method uses a pure greedy procedure to move a node between two different sets. The detected clusters are also core-attachment structures. In particular, GSM-CA firstly defines seed edges of the core from the neighbourhood graphs based on the highest weight and then detects protein-complex cores as the hearts of protein complexes. Finally, GSM-CA includes attachments into these cores to form biologically meaningful structures. The new algorithm is applied to the protein interaction network of S. cerevisiae. The modules identified by the new algorithm are mapped to the MIPS [22] benchmark complexes and validated by GO [23] annotations. The experimental results show that the identified modules are statistically significant. In terms of prediction accuracy, the GSM-CA method outperforms several other competing algorithms. Moreover, most of the previous methods can not detect the overlapping functional modules by generating separate subgraphs. But GSM-CA can not only generate non-overlapping clusters, but also overlapping clusters.

The GSM-CA method achieves high accuracy. However, it is computationally expensive. Many module detection approaches are based on the traditional hierarchical methods, which is also computationally inefficient because the hierarchical tree structure generated by the repeated computational process cannot provide adequate information to identify whether a network belongs to a module structure or not. To further improve the computational process of these module detection approaches, the Greedy Search Method based on Fast Clustering (GSM-FC) is proposed in this paper. The edge weight based GSM-FC method uses the greedy procedure to traverse all edges just once to separate the network into the suitable sets of modules. The experimental results demonstrate that the newly proposed algorithm can reduce the computational time noticeably while maintaining high prediction accuracy compared to GSM-CA.

Briefly then, the outline of this paper is as follows. In Section 2 the implementation of our two methods are described in details. In Section 3, our algorithm is applied to the protein interaction network of S. cerevisiae yeast and the results are analyzed. In Section 4, the conclusions are given.

## Methods

### Definitions

Protein interaction networks can be represented as an undirected graph *G *= (*V *, *E*), where V is the set of vertices and *E *= {(*u*,*v*)| *u*,*v *∈ *V*} is the set of edges between the vertices. For a node *v *∈ *V *, the set of v's direct neighbours is denoted as *N_v_*. *N_v _*is defined as *N_v _*= {*u*| *u *∈ *V*,(*u*,*v*) ∈ *E*}. Before introducing details of the algorithm, some terminologies used in this paper are defined.

The closeness *cn*_*nk *_of any node n with respect to some node k in cluster c is defined by (1).

(1) $$cn_{nk}=\frac{\left|NC_{n}\cap NC_{k}\right|}{\left|NC_{k}\right|}$$

Here, *NC_n _*is the set of n's direct neighbours in cluster c, and *NC_k _*is the set of k's direct neighbours in cluster c.

The DPClus algorithm defines the weight *w_uv _*of an edge (*u*,*v*) ∈ *E *as the number of the common neighbours of the nodes u and v. It is likely that two nodes that belong to the same cluster have more common neighbours than two nodes that do not. For two edges having the same number of common neighbours, the one that has more interactions between the common neighbours is more likely to belong to the same cluster.

Therefore, the definition of *w_uv _*is modified in the paper by (2)

(2) $$w_{uv}=|N_{uv}|+\alpha *|E_{uv}|$$

Here *N_uv _*= *N_u _*∩ *N_v_*, *E_uv _*= {(*v_j_*, *v_k_*)| (*v_j_*, *v_k_*) ∈ *E*, *v_j_*, *v_k _*∈ *N_uv_*} and *α *is the interaction factor to indicate how important the interactions are. *α*'s default value is set as 1.

The number of common neighbours between any two nodes is actually equal to the number of paths of length 2 between them. This definition of weight is used to cluster the graphs that have densely connected regions separated by sparse regions. In relatively sparse graphs, the nodes on the path of edges with length 3 or length 4 can be considered.

The highest edge weight of a node n is defined as *hw_n _*= max (*w_nu_*) for all u such that (*n*, *u*) ∈ *E*. The highest weight edge (n, v) of node n is the edge satisfying the condition that *w_nv _*= *hw_n_*.

### Greedy Search Method based on Core Attachment structure (GSM-CA)

Because core and peripheral proteins may have different roles and properties due to their different topological characteristics, a Greedy Search Method based on Core Attachment structure called GSM-CA is proposed based on the definition of the edge weight and two evaluation criterion for judging a node as a core node or an attachment node. GSM-CA uses a greedy procedure to get the suitable set of clusters. It first generates the core of a cluster, and then selects reliable attachments cooperating with the core to form the final cluster. The algorithm is divided into six steps: 1) Input & initialization; 2) Termination check; 3) Seed selection; 4) Core formation; 5) Attachments selection; 6) Output & update. The functional modules are determined by final clusters. The whole description of the GSM-CA algorithm is shown in the following.

### Input & initialization

The input to the algorithm is an undirected simple graph and hence the associated matrix of the graph is read first. The user need decide the minimum value for closeness in cluster formation. The minimum value will be referred to as *cn_in_*. Each edge's weight is computed based on formula (2). It is computed just once and will not be recalculated in the following steps.

### Termination check

Once a cluster is generated, it is removed from the graph. The next cluster is then formed in the remaining graph and the process goes on until no seed edge whose weight is above one (i.e. *w_uv _*> 1) can be found in the remaining graph.

### Seed selection

Each cluster starts at a deterministic edge called the seed edge. The highest weight edge (n, v) of node n satisfying the condition that *w_nv _*=*hw_n _*is considered as the seed edge in the remaining graph.

### Core formation

A protein complex core is a small group of proteins which show a high co-expression patterns and share high degree of functional similarity. It is the key functional unit of the complex and largely determines the cellular role and essentiality of the complex [21,26-28]. For example, a protein in a core often has many interacting partners and protein complex cores often correspond to small, dense and reliable subgraphs in PPI networks [28].

The core starts from a single edge and then grows gradually by adding nodes one by one from the neighbours. The neighbours of a core are the nodes connected to any node of the core but not part of the core. The core is referred to as C. For a neighbour u of C, if u's neighbour v linked by u's highest weight edge (u, v) is in C, u is considered to be included into the core. Before including u to C, the condition, *cn_uv _*>= *cn_in _*, is checked and the neighbour whose highest edge weight is largest is included. This process goes on until no such neighbour can be found, and then the core of one cluster is generated.

### Attachments selection

After the core of one cluster has been detected, the peripheral information of each core is extracted and reliable attachments cooperating with it are selected to form the final cluster. For each neighbour u of the core C, if u's neighbour v linked by u's highest weight edge (u, v) is in C, $\frac{\left|V_{uv}\right|}{\left|N_{uv}\right|}$ is computed. *V_uv _*is the common neighbours of u and v in the core C. *N_uv _*is the common neighbours of u and v in graph G.

If $\frac{\left|V_{uv}\right|}{\left|N_{uv}\right|}>0.5$, u will be selected as an attachment. After all neighbours of the core are checked, the final cluster is generated.

### Output & update

Once a cluster is generated, graph G is updated by removing the present cluster. The nodes belonging to the present cluster and the incident edges on these nodes are marked as clustered and not considered in the following. Then in the remaining graph, each node's highest edge weight is updated by not considering the edges that have been marked. The pseudocode of the GSM-CA algorithm is shown in Table 1.

**Table 1:** 

Algorithm GSM-CA
Input: a graph *G *= (*V *, *E*), parameters *cn_in _*; Output: identified modules; (1) Compute the edge weight **For **each edge *e*(*u*,*v*) ∈*E ***do** compute *w_uv_*; **End For** (2) Form core Select the edge *e*(*u*,*v*) with highest weight in *G*; **If ***w_uv _*< 1 **then **exit; **End If** Initial core *C *={*u*,*v*}; **While **neighbour *i *whose neighbor *j *linked by *i's *highest weight edge is in *C *and *cn_ij _*≥ *cn_in _***do** *C*. add *(i)*; **End While** (3) Select attachments for core **For each **neighbor *u *of the core *C ***do** **If ***u's *neighbor *v *linked by *u's *highest weight edge is in ***C*** and $\frac{\left|V_{uv}\right|}{\left|N_{uv}\right|}>0.5$**then** *u *is selected as attachment of *C* **End If** **End For each** (4) Output results and update the highest edge weight Output *C *and delete *C *from *G*, update each vertex's highest edge weight in the remaining *G* (5) Repeat from step 2 to step 4, until reaching the termination condition of step 2.

### Generation of overlapping clusters

In the above algorithm, once a cluster is generated it is marked as clustered and not considered in the following, and the next cluster is generated in the remaining graph. Therefore, non-overlapping clusters are generated. In order to generate overlapping clusters, the existing non-overlapping clusters are extended by adding nodes to them from their neighbours in the original graph (considering the marked nodes and edges). Then in the original graph excluding the edges between the nodes that have been marked as clustered, each node's highest edge weight is updated.

### Greedy Search Method based on Fast Clustering (GSM-FC)

Many module detection approaches including GSM-CA is computationally expensive. The traditional hierarchical tree structure generated by these approaches can't provide adequate information to identify which subtree belongs to a module structure. As a result, the module structure need be evaluated repeatedly based on the module definition. During the computational process, the edge weight of neighbouring nodes need be recomputed after one edge is deleted. The edge weight calculation is based on the shortest path between vertices. Since the shortest path problem has high time complexity, these approaches are even not scalable for the networks with the medium size.

The GSM-FC can avoid repeated module structure evaluation because the module structure can be identified based on inherent network organization and the greedy algorithm. The GSM-FC traverses all edges once then generates the clusters. Moreover, the GSM-FC utilizes properties of subnetworks, which can reflect the network topology more effectively. As a result, the computational efficiency of the GSM-FC method can be improved noticeably.

The GSM-FC algorithm is divided into three steps: 1) Input & initialization; 2) Cluster formation; 3) Output.

### Input & initialization

The input to the algorithm is an undirected simple graph and hence the associated matrix of the graph is read first. Each edge's weight is computed based on formula (2). All vertices in the graph G are initialized as singleton clusters at first step.

### Cluster formation

During this step, all edges are traversed gradually and a greedy procedure is used to assemble the nodes into clusters. For one edge (u, v), if u and v are not in the same cluster, they are considered to be merged. If the edge weight *w_uv _*is u's highest edge weight, and then an edge from u to v is added in order to merge the cluster including u into the cluster including v. Similarly, if the edge weight *w_uv _*is v's highest edge weight, and then an edge between v and u is added in order to merge the cluster including v with the cluster including u. If the edge weight *w_uv _*is neither u's highest edge weight nor v's highest edge weight, the edge (u, v) is ignored and the next edge is evaluated.

### Output

After all edges have been visited, the subnetworks generated during the cluster merging process are outputted. These subnetworks are considered as modules. The pseudocode of GSM-FC algorithm is shown in Table 2.

**Table 2:** 

Algorithm GSM-FC
Input: a graph *G *= (*V *, *E*); Output: identified modules; (1)**For **each edge *e*(*u*,*v*) ∈*E ***do** compute *w*_*uv *_; add *e*(*u*,*v*) to queue *S*_*q*_ **End for** (2)**While ***Sq *≠ ∅ **do**; *e*(*u*, *v*) ← *S*_*q*_; **If ***L*(*u*) ≠ *L*(*v*) **then **//L is cluster label *i *= *L*(*u*); *j *=*L *(*v*); **If ***w_uv _*==*hw_u _*|| *w_uv _*== *hw_v _***then** *V *(*C_i_*) = *V *(*C_i_*) ∪ *V*(*C_j_*); //*C *represents the cluster **End if** **End if** **End while**

### Efficiency analysis

Compared to the other algorithms, the advantage of the GSM-FC algorithm are computationally efficient. The GSM-FC algorithm just needs to visit all edges once without any parameter input. The time complexity of the clustering process is linear. The edge weight calculation is the most time-consuming step for the clustering process. Let n and m denote the number of vertices and edges in a protein interaction network respectively; k be the average number of neighbours of all the vertices, i.e. $k=\frac{1}{n}\sum_{v\in V}|N_{v}|$; Then, the complexity of calculating all the edge clustering coefficients is *O*(*k*^2^*m*). Since the time complexity of the hierarchical merging process is *O*(*m*), the total time complexity of the GSM-FC algorithm is *O*(*k*^2^*m*). In general, k is much smaller than the number of vertices n and can be considered as a constant because it is well known that the protein interaction network is scale-free, in which most proteins only participate in a small number of interactions [31].

## Experimental setup and result analysis

### Data set and the criterion of performance evaluation

In order to evaluate effectiveness of the new system, our algorithm is applied to the full DIP (the Database of Interacting Proteins) [24] yeast dataset, which consists of 17201 interactions among 4930 proteins [21]. It is more complex and difficult to identify the modules using the full dataset than using the core dataset. The performance of our method is compared with several competing algorithms including MCODE, CFinder, DPClus, and COACH. The parameter selection for these algorithms is based on authors' recommendation. Several metrics including f-measures and p-value are used for rigorous performance evaluation.

The experimental results are based on a reference dataset of known yeast protein complexes retrieved from the MIPS [22]. While it is probably one of the most comprehensive public datasets of yeast complexes available up to date, it is by no means a complete dataset--there are still many yeast complexes that need to be discovered. After filtering the predicted protein complexes and complexes composed of a single protein from the dataset, a final set of 214 yeast complexes are used as our evaluation benchmark.

The overlapping score [17] between a predicted complex and a real complex in the benchmark, *OS*(*p*, *b*) = *i*^2^/(p*b), is used to determine whether these complexes match with each other, where i is the size of the intersection set of a predicted complex with a known complex, p is the size of the predicted complex and b is the size of the known complex. If *OS*(*p*, *b*) ≥ *ω*, they are considered to be matching (*ω *is set as 0.20 which is adopted in the MCODE paper [17]). We assume that P is the sets of complexes predicted by a computational method and B is the sets of target complexes in the benchmark respectively. The set of True Positives (TP) is defined as *TP *= {*p*|*p *∈ *P*, ∃*b *∈ *B*, *OS*(*p*, *b*) ≥ *ω*}, while the set of False Negatives (FN) is defined as *FN *= {*b*|*p *∈ *P*, *b *∈ *B*, ∀ *p*(*OS*(*p*, *b*) < ω)}. The set of False Positives (FP) is *FP *= *P *- *TP *, while the set of known benchmark complexes matched by predicted complexes (TB) is *TB *= *B *- *FN*. The sensitivity and specificity [17] are defined as:

(3) sensitivity=|TP|/(|TP|+|FN|)

(4) specicity=|TP|/(|TP|+|FP|)

S-measure, as the harmonic mean of sensitivity and specificity, can be used to evaluate the overall performance of the different techniques.

(5) $$s-measure=2^{*}sensitivity^{*}specificity/(sensitivity+specificity)$$

P-values are used to evaluate the biological significance of our predicted complexes. P-values represent the probability of co-occurrence of proteins with common functions. Low p-value of a predicted complex generally indicates that the collective occurrence of these proteins in the module does not happen merely by chance and thus the module has high statistical significance. In our experiments, the p-values of complexes are calculated by the tool called SGD's Go::TermFinder [23]. SDG's Go: TermFinder uses all the three types of ontology including Biological Process (BP), Molecular Function (MF) and Cellular Component (CC). The cutoff of the p-value is set as 0.01. The average -log(*p*-*value*) of all modules is calculated by mapping each module to the annotation with the lowest p-value.

Let the total number of proteins be N with a total of M proteins sharing a particular annotation. The p-value of observing m or more proteins that share the same annotation in a cluster of n proteins, using the Hyper-geometric Distribution is defined as (6):

(6) $$p-value=\overset{n}{\underset{i=m}{\sum }}\frac{\left(\begin{array}{c} M\\ i \end{array}\right)\left(\begin{array}{c} N-M\\ n-i \end{array}\right)}{\left(\begin{array}{c} N\\ n \end{array}\right)}$$

The average f-measure is used to evaluate the overall significance of each algorithm. f-measure of an identified module is defined as a harmonic mean of its recall and precision [25].

(7) $$f-measure=\frac{2^{*}recall^{*}precision}{recall+precision}$$

(8) $$recall=\frac{\left|M\cap F_{i}\right|}{\left|F_{i}\right|}$$

(9) $$precision=\frac{\left|M\cap F_{i}\right|}{\left|M\right|}$$

Where *F*_*i *_is a functional category mapped to module M. The proteins in functional category *F_i _*are considered as true predictions, the proteins in module M are considered as positive predictions, and the common proteins of *F_i _*and M are considered as true positive predictions. Recall is the fraction of the true-positive predictions out of all the true predictions, and precision is the fraction of the true positive predictions out of all the positive predictions [25]. The average f-measure value of all modules is calculated by mapping each module to the function with the highest f-measure value.

### Experimental results for GSM-CA method

Table 3 compares results obtained by several popular methods with MIPS benchmark complexes. Table 3 indicates that the number of correctly predicted complexes using MCODE, CFinder, DPClus and GSM-CA is less than the number of benchmark complexes matched by predicted complexes. But COACH is opposite. Because COACH detects the clusters from each node, the overlapping rate is high. Although the redundancy-filtering procedure is used, some predicted complexes are still similar and match the same benchmark complex. Table 3 indicates that the s-measure of COACH (0.307) is highest among the methods of MCODE, CFinder and DPClus. The s-measure of GSM-CA (0.380) is significantly higher than that of COACH. In addition, the overall performance of COACH is much better than CoreMethod[21] which is another approach based on core-attachment structure.

**Table 3:** Results of various algorithms compared with MIPS complexes using DIP data

Algorithms	MCODE	CFinder	DPClus	COACH	GSM-CA
#predicted	59	245	1143	745	**353**
complexes					
|TP|	18	52	133	155	**105**
|TB|	19	61	144	106	**119**
s-measure	0.132	0.231	0.198	0.307	**0.380**

**Figure 1:**
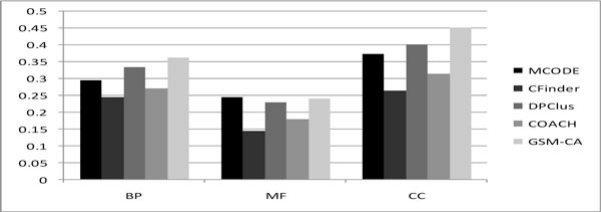
**Comparison of f-measure based on three types of GO of GSM-CA and other algorithms**.

Comparison of the results before and after adding attachments is shown in Table 4. The comparison shows that after adding attachments, the average size of modules grows from average size of 5.29 into 7.37. Moreover, the f-measure of BP and -log(*p-value*) have improved noticeably after adding attachments. All of these indicate that protein complexes indeed contain Core/attachment structures. Comparisons of biological significance of modules predicted by several algorithms are shown in Table 5. MCODE is not considered since it just generates a small number of modules. Table 5 indicates that the proportion of significant modules predicted by GSM-CA is highest and -log(*p-value*) of GSM-CA is also higher than the other algorithms. Moreover, in all of the other methods, the average f-measure of DPClus is highest (0.335), however, the average f-measure of GSM-CA is 0.362, which is higher than that of DPClus. The detailed comparison of f-measure based on all the three types of Gene Ontology (GO) Terms including Biological Process, Molecular Function, and Cellular Component is shown in Figure 1. Figure 1 indicates that the average f-measure of Cellular Component GSM-CA is also highest (0.453) in all of the methods.

**Table 4:** Comparison of the results before and after adding attachments

	Average Size	f-measure of BP	-log(p-value)
Before	5.29	0.356	7.2
**After**	**7.37**	**0.362**	**8.6**

**Table 5:** Statistical significance of functional modules predicted by various methods

Algorithms	No. of Modules size>=3	No. of Significant Modules	Average Size	Maximum	f-measure of BP	-log(p-value)	Parameters
MCODE	59	54	83.8	549	0.296	10.87	fluff = 0.1; VWP = 0.2
CFinder	245	157	10.2	1409	0.246	4.49	K = 3
DPClus	217	187	5.23	25	0.335	6.78	Density = 0.7;CP_in_=0.5
COACH	746	608	8.54	44	0.272	6.96	Null
**GSM-CA**	**187**	**168**	**7.37**	**79**	**0.362**	**8.6**	**CN_in_=0.5**
**GSM-FC**	**113**	**106**	**9.65**	**118**	**0.359**	**10.46**	**Null**

Table 6 lists the top 10 most significant modules identified by the GSM-CA method. They are sorted in the increasing order based on the p-value.

**Table 6:** List of top ten scoring modules identified by GSM-CA and their most enriched GO terms for Biological Process

ID	Size of module	Number of proteins enriched the same GO Term	Size of GO Term	Name of GO Term	p-value
1	35	33	221	rRNA processing	1.70e-41
2	42	38	323	ribosome biogenesis	1.61e-39
3	13	13	15	tRNA transcription	1.92e-35
4	19	14	19	mRNA polyadenylation	1.96e-31
5	11	11	12	cyclin catabolic process	8.92e-31
6	14	12	14	polyadenylation-dependent snoRNA 3'-end processing	1.91e-30
7	19	18	93	mitochondrial translation	8.08e-30
8	16	14	29	energy coupled proton transport, down electrochemical gradient	1.33e-29
9	22	14	20	RNA polymerase II transcriptional preinitiation complex assembly	2.70e-29
10	19	18	101	nuclear mRNA splicing, via spliceosome	8.91e-29

**Figure 2:**
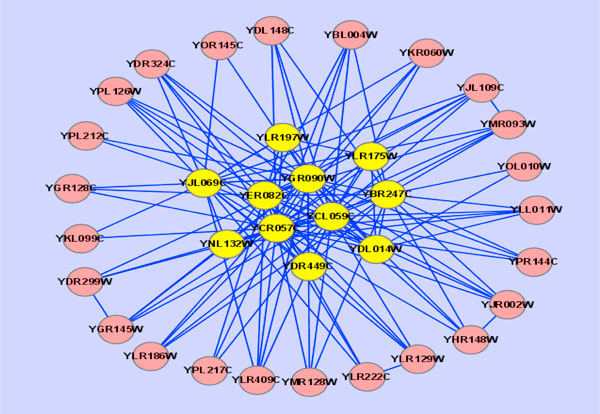
**An example of modules identified by the GSM-CA method**.

Figure 2 visualizes the structure of the modules identified by the GSM-CA method. The yellow nodes form the core and the red nodes represent the attachments.

**Figure 3:**
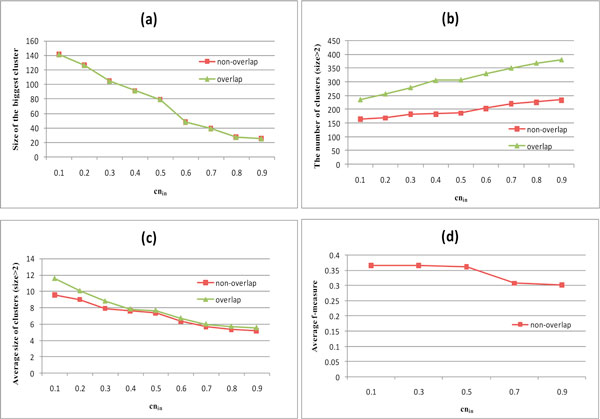
**The effects of cn_in_ on clustering**. (a) The size of the biggest cluster (b) The total number of the clusters whose size is greater than 2 (c) The average size of the clusters whose size is greater than 2 (d) The average f-measure.

The GSM-CA method used the parameter *cn_in _*, and the effects of changing the parameter *cn_in _*for cluster generation are shown in Figure 3. When *cn_in _*changes from 0.1 to 0.9, the size of the biggest cluster and the average size of clusters decrease but the number of cluster increases. The sizes of the biggest overlapping clusters are same as that of the non-overlapping clusters, so Figure 3(a) just draws one line. In Figure 3(b), the total number of the overlapping clusters is more than that of the non-overlapping clusters. In Figure 3(c), the average size of the overlapping clusters is bigger than that of the non-overlapping clusters. The effect of *cn_in _*on f-measure is shown in Figure 3(d). Figure 3 indicates that the f-measure is relatively lower when *cn_in _*>0.5. Because when *cn_in _*is close to 1, the core of cluster is almost clique. It may be too strict to match well with the known annotations. The f-measure is basically stable when *cn_in _*<= 0.5. So *cn_in _*is set as 0.5.

### Experimental results for the GSM-FC method

Table 7 compares the running time of the GSM-FC method with that of other functional module identification algorithms. These algorithms are applied to the full DIP yeast dataset, which consists of 17201 interactions among 4930 proteins. Table 7 shows that the running time for the GSM-FC method is shortest since it just visits all edges once. Since COACH detects cores from each vertex in the network once, the running time for COACH is also small, but it is greater than the running time of GSM-FC. CFinder uses an efficient method called CPM to detect maximum cliques, so it is not time-consuming. DPClus needs many sorting and computing, so it is computationally costly.

**Table 7:** Comparison of the running time of the GSM-FC algorithm and other algorithms

Algorithms	The running time
MCODE	71.5 s
COACH	6.8 s
CFinder	24.4 s
DPClus	926.0 s
GSM-AC	82.6 s
**GSM-FC**	**3.4 s**

Table 5 compares the biological significance of modules predicted by several algorithms. The improved average f-measure and -log(*p*-*value*) demonstrate that the modules identified by the GSM-FC method have higher statistical significance than other methods. The GSM-FC method generates fewer numbers of clusters with the bigger average cluster size compared to the GSM-CA method. Both Table 5 and Table 7 show that the GSM-FC method can reduce the computational time noticeably while keeping high prediction accuracy compared to GSM-CA. Furthermore, the GSM-FC method which doesn't require any input parameters can be applied to even larger protein interaction networks.

## Conclusions

Identification of functional modules is crucial to the understanding of the structural and functional properties of protein interaction networks. The increasing amount of protein interaction data has enabled us to detect protein functional modules. In this paper, a Greedy Search Method based on Core-Attachment structure called GSM-CA is proposed to mine functional modules from the protein interaction networks. Because core and peripheral proteins may have different roles and properties due to their different topological characteristics, the GSM-CA method defines edge weight and two criterion for determining core nodes and attachment nodes. It first generates the core of a module, and then forms the module by including attachments into the core. The GSM-CA method is applied to the typical PPI networks of S. cerevisiae. The MIPS benchmark and the GO annotation are used to validate the identified modules and compare the performances of our algorithm with several other algorithms including MCODE, CFinder, DPClus, and COACH. The evaluation and analysis show that most of the functional modules predicted by our algorithm have high functional similarity and match well with the benchmark. The quantitative comparisons reveal that our algorithm outperforms the other competing algorithms. Many module detection approaches utilize the traditional hierarchical clustering methods, which are computationally costly because the tree structure produced by the hierarchical clustering methods can not provide adequate information to identify whether a network belongs to a module structure or not. To overcome these problems, the Greedy Search Method based on Fast Clustering (GSM-FC) is proposed. The GSM-FC method takes advantages of the greedy search procedure to separate the network into the suitable set of modules. The experimental results show that the GSM-FC method can reduce the computational time significantly while keeping high prediction accuracy compared to GSM-CA. For the future work, the algorithm need be applied to the weighted graph. How to incorporate diverse biological information into the explorative analysis of protein complexes in PPI networks is another interesting question for further research.

## Competing interests

The authors declare that they have no competing interests.

## Authors' contributions

JH supervised the work, and JH, BY and WZ contributed to the problem formulation and paper writing. JH and CL conducted research on the algorithms of GSM-CA and GSM-FC, and CL developed and implemented the algorithms. The manuscript was drafted by JH and CL. All authors read and approved the final manuscript.
